# Leveraging microbiome rediversification for the ecological rescue of soil function

**DOI:** 10.1186/s40793-023-00462-4

**Published:** 2023-01-23

**Authors:** William L. King, Sarah C. Richards, Laura M. Kaminsky, Brosi A. Bradley, Jason P. Kaye, Terrence H. Bell

**Affiliations:** 1 Department of Plant Pathology and Environmental Microbiology, The Pennsylvania State University, 317 Buckhout Lab, University Park, PA 16802 USA; 2 School of Integrative Plant Science, Cornell University, Ithaca, NY 14853 USA; 3 Intercollege Graduate Degree Program in Ecology, The Pennsylvania State University, University Park, PA 16802 USA; 4 Intercollege Graduate Degree Program in International Agriculture and Development, The Pennsylvania State University, University Park, PA 16802 USA; 5 Department of Ecosystem Science and Management, The Pennsylvania State University, University Park, PA 16802 USA

**Keywords:** Microbial function, Biodiversity, Nitrification, Community inoculum, Microbial establishment

## Abstract

**Background:**

Global biodiversity losses threaten ecosystem services and can impact important functional insurance in a changing world. Microbial diversity and function can become depleted in agricultural systems and attempts to rediversify agricultural soils rely on either targeted microbial introductions or retaining natural lands as biodiversity reservoirs. As many soil functions are provided by a combination of microbial taxa, rather than outsized impacts by single taxa, such functions may benefit more from diverse microbiome additions than additions of individual commercial strains. In this study, we measured the impact of soil microbial diversity loss and rediversification (i.e. rescue) on nitrification by quantifying ammonium and nitrate pools. We manipulated microbial assemblages in two distinct soil types, an agricultural and a forest soil, with a dilution-to-extinction approach and performed a microbiome rediversification experiment by re-introducing microorganisms lost from the dilution. A microbiome water control was included to act as a reference point. We assessed disruption and potential restoration of (1) nitrification, (2) bacterial and fungal composition through 16S rRNA gene and fungal ITS amplicon sequencing and (3) functional genes through shotgun metagenomic sequencing on a subset of samples.

**Results:**

Disruption of nitrification corresponded with diversity loss, but nitrification was successfully rescued in the rediversification experiment when high diversity inocula were introduced. Bacterial composition clustered into groups based on high and low diversity inocula. Metagenomic data showed that genes responsible for the conversion of nitrite to nitrate and taxa associated with nitrogen metabolism were absent in the low diversity inocula microcosms but were rescued with high diversity introductions.

**Conclusions:**

In contrast to some previous work, our data suggest that soil functions can be rescued by diverse microbiome additions, but that the concentration of the microbial inoculum is important. By understanding how microbial rediversification impacts soil microbiome performance, we can further our toolkit for microbial management in human-controlled systems in order to restore depleted microbial functions.

**Supplementary Information:**

The online version contains supplementary material available at 10.1186/s40793-023-00462-4.

## Background

Biodiversity is critical to the stability and efficiency of many ecosystem functions, as it can correlate directly with certain ecosystem services and can provide functional insurance under fluctuating environmental conditions [1–4]. However, global biodiversity is decreasing due to numerous interacting forces, including land use modification, habitat loss, and climate change [5, 6]. This has led to efforts to restore ecosystem function in various systems following environmental degradation [7].

In managed soils, such as farming systems, human activities often deplete soil microbial biodiversity thereby impacting microbially-mediated soil functions [8–14]. For instance, agricultural nutrient additions can deplete taxa that play critical roles in nutrient cycling, weaken mutualistic plant–microbe interactions, and reduce microbial diversity overall [8–12]. Residues from synthetic inputs (e.g. chemical fertilizers) can also have long-lasting effects on microbial biomass and the abundance of important nutrient solubilizers, such as mycorrhizal fungi, even 20 years post-management [15]. This depletion in microbial diversity and function in agricultural soils has led to increased interest in managing microorganisms in order to augment or restore ecosystem functions of interest.

One such function of interest in agricultural systems is nitrification. Nitrification is a multi-step process which involves the conversion of ammonia to nitrite by ammonia-oxidizing bacteria (AOB) and archaea (AOA), and the conversion of nitrite to nitrate by nitrite-oxidizing bacteria (NOB) [16]. Nitrogen is often a limiting factor for crop growth and farmers apply synthetic fertilizers (i.e. ammonium) to augment plant-available N. Plants can freely take up nitrogen in the form of ammonium and nitrate, but high concentrations of ammonium can cause toxicity and suppress plant growth [17, 18], while nitrate is more susceptible to being leached into surrounding environments [19, 20]. As plant growth is often the most optimal when both ammonium and nitrate are available, and because of ammonium toxicity, there is interest in not completely disrupting nitrification. Of particular interest is the sensitivity of nitrification to microbial diversity loss. Nitrifiers are often rare and their functions are typically additive. While some nitrifiers are able to perform all of the nitrification steps (i.e. COMMAMOX) [21, 22], their contributions are additive to the overall system performance.

Although we are far from a consensus on how to apply microbial management in agriculture, it is increasingly common [23] and can take many forms [24]. Management may be active, through the targeted introduction of specific beneficial microorganisms, or passive, by altering practices to leverage existing resident microorganisms or those that may enter the system through passive dispersal [25, 26]. Active management can augment functions of interest (i.e. enhanced P mineralization), but microbial establishment and functional performance in novel recipient environments is often unreliable due to various abiotic and biotic constraints [25–27]. Passive management can include increasing crop diversity and/or retaining natural lands that may provide reservoirs for diverse microbial influx [28]. Since many soil functions are provided by the combined contributions of many microbial taxa rather than the outsized impacts of a few, there may be value in the rediversification of whole microbial assemblages that have experienced biodiversity loss. In human systems, for instance, microbiome rediversification of the colon has been successfully used as a treatment for diseases such as *Clostridium difficile* infection [29]. However, the successful application of microbiome rediversification in soils is less clear and the origin of the soil inoculum could be important for determining aboveground plant diversity [30].

A previous study by Calderon et al. [31] aimed to test the efficacy of microbiome rediversification (described in the paper as *ecological rescue*) in arable soils. By recolonizing a sterilized soil using a serially diluted source soil (i.e. 10^0^, 10^−4^, 10^−6^ and 10^−8^), they demonstrated that soil microbial diversity loss led to nitrification disruption. The chosen sterilized soil was described as an intermediary soil, as it was abiotically distinct from the starting source soils to avoid a “home-field advantage” for the applied microbes [31]. To investigate whether microbiome rediversification could rescue diversity and function, diluted source soil microbiomes (i.e. 10^−4^ and 10^−6^) were introduced into the recolonized intermediary soils. However, while microbial diversity increased in some treatments, nitrification was not restored. We expected that this lack of functional rescue may have been due to either (1) the impact of novel environmental pressures on the microorganisms introduced to the intermediary soil (i.e. abiotic constraints) or (2) the diversity of reintroduced microbiomes was not sufficiently high enough to replenish soil function (i.e. biotic constraints).

Building upon the work of Calderón et al. [31], we chose to isolate the biotic constraints of microbiome rediversification by recolonizing a sterilized soil with its own corresponding soil microorganisms (i.e. no intermediate soil) to reduce the impact of abiotic constraints on microbial establishment. We performed two experiments: (1) a dilution-to-extinction experiment to identify how depleting microbial diversity impacts nitrification, and (2) a microbiome rediversification experiment to reintroduce diversity to the nitrification disrupted microcosms (i.e. 10^−6^) from the first experiment. All microcosms used for the microbiome rediversification experiment were initially recolonized with a low diversity microbiome (i.e. 10^−6^) to identify whether introduced diversity would be impacted by prior colonization and cause a regime shift, and whether function could be restored through microbiome rediversification. Importantly, we employ a water control as a reference to parse the influence of diversity and biotic constraints on microbiome development. We chose to quantify the impact of microbiome rediversification on nitrification as it is an additive function and corresponds to the functions measured by Calderón et al. [31]. We used an agricultural and a forest soil to contrast physiochemically distinct soils with different management practices, for example agricultural soils are expected to have reduced microbial diversity and biased towards copiotrophs. We hypothesized that: (1) depleting microbial diversity would disrupt nitrification, (2) microbiome rediversification would restore nitrification, (3) microbial composition would cluster according to nitrification restoration and the diversity of reintroduced microorganisms, and (4) increasing the richness of added microorganisms would cause a stronger regime shift. Understanding the relationship between the diversity of microbial additions and the rescue of microbial function in soil will enhance our toolkit for microbial management in microbially-depleted soils.

## Methods

### Soil collection and preparation

To assess functional recovery in soils differing in both land use and microbial composition, we collected two soils (top 10 cm) for this experiment: (1) an agricultural soil from an organic certified research farm at the Pennsylvania State University (PSU) Russell E. Larson Agricultural Research Center (40° 43′ 16.1″ N 77° 55′ 42.9″ W) and (2) a forest soil from the Pennsylvania State University managed contiguous forest stemming from Rothrock State Forest (40° 42′ 45.9″ N 77° 55′ 53.4″ W). Each collected soil was sieved through a 2.0 mm wire mesh and split into three portions, with the first two either: (1) stored at room temperature in a sterile container, for use as a source soil for generating initial soil slurry inoculum, or (2) immediately frozen, for use in the microbiome rediversification experiment (see *Microbiome rediversification to restore nitrification*). The remaining sieved soil was air dried and sterilized by autoclaving three individual times with 24 h between each autoclaving cycle. Sterile soil was analyzed by the Agricultural Analytical Services Laboratory at Pennsylvania State University (Additional file 1: Table S1). The sterile agricultural (farm) soil was identified as a silt loam textural class (sand: 18.9%; silt: 56.3%; clay: 24.8%), while the sterile forest soil was identified as a sandy loam textural class (sand: 65.0%; silt: 25.0%; clay: 10.0%).

### Soil microcosm setup, serial dilutions and inoculation

**Fig. 1:**
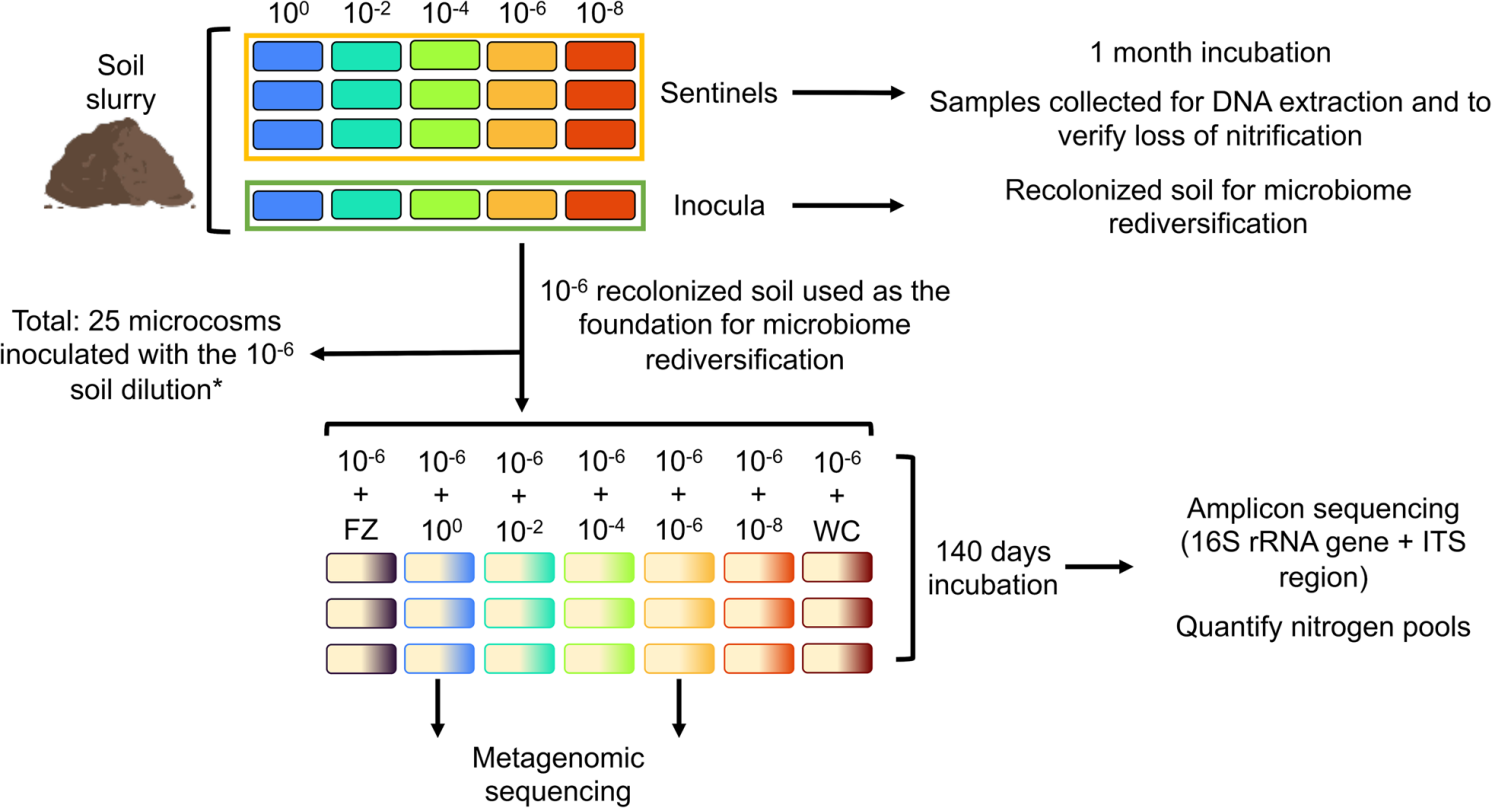
Experimental design schematic. *10^−6^ was chosen as the foundational microcosms to correspond to the N-cycling functional loss observed in Calderón et al. [31]. FZ and WC represent the frozen soil and water control, respectively (see Microbiome rediversification to restore nitrification)

To evaluate the impact of microbial diversity loss on soil functional disruption and recovery, we used soil microcosms in which sterilized soil was recolonized by serially diluted soil slurries. This dilution-to-extinction approach is a common approach for reducing microbial diversity in culture-independent systems [32]. Soil microcosms were built by first placing 30 g of sterile soil in sterile Petri dishes. To make the soil slurry inocula, 100 g of equivalent dry mass of the non-sterile sieved soil was mixed with 150 mL of sterile water (1:1.5 ratio) and blended at 22,000 rpm using a 70% ethanol and UV sterilized blender to make a soil slurry. The resultant slurry was collected (10^0^ dilution) and a portion was serially diluted to 10^−2^, 10^−4^, 10^−6^, and 10^−8^ with sterile water. Soil microcosms were inoculated to 70% of the estimated water holding capacity for each soil with the respective soil slurry dilution. For each serial dilution (i.e. 10^0^, 10^−2^, 10^−4^, 10^−6^, and 10^−8^), we inoculated four soil microcosms (Fig. 1) and soil microcosms were allowed to incubate for one month in a humidity chamber maintained at 75% humidity and 20 °C. Three of the soil microcosms were used as “sentinel microcosms” to assess disruption of nitrification due to dilution, while the remaining soil microcosm was used as an inoculum source in the microbiome rediversification experiment. For the 10^−6^ inoculated microcosms, we inoculated an additional 21 soil microcosms per soil to act as our microbiome base (henceforth known as foundational microcosms) for the microbiome diversification experiment. The 10^−6^ microcosms were chosen because we observed nitrification disruption (see Results). In total, we inoculated 82 microcosms across the two soils (for each soil: 15 sentinel microcosms + 5 inoculum microcosms + 21 foundation microcosms for the microbiome rediversification experiment). Following a month of incubation, aliquots from each sentinel microcosm were collected for DNA extraction and a portion used for quantifying pools of ammonium and nitrate.

### Microbiome rediversification to restore nitrification

After soil microcosm incubation, a recolonized soil microcosm from each serial dilution was used as an inoculum source in the microbiome rediversification experiment (shown as “Inocula” in Fig. 1). Inocula were made by mixing the entire soil microcosm (approximately 30 g of equivalent dry mass soil) with 45 mL of sterile water in an ethanol- and UV-sterilized container. A slurry was made by blending the soil and water with a hand blender sterilized with 70% ethanol. For each inoculum, 2 mL of slurry was used to inoculate three replicate foundational microcosms (i.e. 10^−6^ recolonized soil microcosms). In addition to the diversity level treatments (i.e. 10^0^, 10^−2^, 10^−4^, 10^−6^, and 10^−8^), we included two controls: (1) an add-back control using frozen sieved soil and (2) a water control. We recently showed that frozen soil can be used to reliably recolonize (i.e. yielding similar microbiome composition) sterile soils [33], hence we use the add-back control to account for the effects of dilution (i.e. making the slurry) and the initial recolonization (i.e. if there are recolonization impacts from the sentinel microcosm stage). The water control was made by spiking sterile water into three replicate foundational microcosms and these act as reference microcosms in the absence of any rediversification attempts. The microbiome rediversification microcosms were allowed to incubate for 140 days in a 75% humidity chamber at 20 °C. Henceforth, the sentinel microcosms will be labelled Dil-0, Dil-2, Dil-4, Dil-6 and Dil-8 (i.e. Dilution) corresponding to 10^0^, 10^−2^, 10^−4^, 10^−6^, and 10^−8^ and the microbiome rediversification microcosms will be labelled Rec-Fz, Rec-0, Rec-2, Rec-4, Rec-6, Rec-8 and Rec-WC (i.e. Recolonization) corresponding to the add-back frozen soil, 10^0^, 10^−2^, 10^−4^, 10^−6^, 10^−8^ and the water control. Other studies using a dilution to extinction approach in soils have shown minimal or no impacts on either overall abundance or abundance of specific taxa after 105 days and 6 weeks (42 days) of soil microcosm incubation [31, 34].

### Quantifying nitrification disruption

Inorganic soil nitrogen was assessed following the procedure outlined in Kaye et al. [35]. Briefly, 10 g of fresh soil sampled from the microcosms was mixed with 30 mL of 2 M KCL, shaken for 1 h, and filtered through Whatman grade 1 paper. The resulting extract was analyzed on a Biotek Elx808 microplate reader (Agilent, Santa Clara, CA) for ammonium (NH_4_^+^) and nitrate (NO_3_^−^) using a colorimetric technique based on Berthelot [36] and Greiss [37] reactions, respectively.

### DNA extraction and amplicon sequencing

Soil aliquots (~ 300 mg) taken from each microcosm were subject to DNA extraction using the NucleoSpin 96 Soil DNA extraction kit (Machery-Nagel; catalogue: 740787.2) as per the manufacturer’s instructions. Bacterial and fungal composition were characterized with amplicon sequencing of the 16S rRNA gene (515F and 806R) and fungal ITS region (ITS1F and 58A2R), respectively. The PCR mixes for both reactions were as follows: 12 µL of Platinum II Hot-Start PCR Master Mix, 1.5 µL of each primer (10 µM), 1.5 µL template DNA and 13.5 µL molecular grade water for a final PCR volume of 30 µL. Bacterial 16S rRNA gene PCR cycling conditions were as follows: 3 min at 94 °C, 25 cycles of: 45 s at 94 °C, 60 s at 50 °C and 90 s at 72 °C, and a final elongation step of 10 min at 72 °C. Fungal ITS PCR cycling conditions were as follows: 3 min at 94 °C, 35 cycles of: 20 s at 94 °C, 30 s at 45 °C and 45 s at 72 °C, and a final elongation step of 5 min at 72 °C. The resulting amplicons were cleaned using Mag-Bind TotalPure NGS magnetic beads (Omega Bio-Tek; catalogue: M1378-01). Illumina indexes were added to the cleaned amplicons with the following PCR ingredients: 12.5 µL of Platinum II Hot-Start PCR Master Mix, 2.5 µL of each index (10 µM) and 2.5 µL of sterile water for a final volume of 25 µL. The indexing PCR cycling conditions were as follows: 1 min at 98 °C, 8 cycles of: 15 s at 98 °C, 30 s at 55 °C, and 20 s at 72 °C, and a final elongation step of 5 min at 72 °C. Indexed amplicons were then normalized using the SequalPrep normalization plate kit (ThermoFisher; catalogue: A1051001), pooled, concentrated with a Centrivap micro IR concentrator (Labconco), and purified with a gel extraction using the PureLink quick gel extraction kit (ThermoFisher; catalogue: K210012). The pooled library was sequenced on the Illumina MiSeq sequencing platform (2 × 250 bp) by the Pennsylvania State University Genomics Core Facility (Huck Institutes for the Life Sciences).

### Sequence analysis

Raw demultiplexed 16S rRNA gene and fungal ITS data were processed using the Quantitative Insights into Microbial Ecology (QIIME 2 version 2020.11) pipeline [38]. Briefly, paired-ended 16S rRNA gene and fungal ITS sequences were trimmed and denoised using DADA2, which also removes chimeric sequences [39]. The classify-sklearn qiime feature classifier was used to assign taxonomy against the Silva v138 [40] or UNITE v8.2 (04.02.2020) database [41] at the single nucleotide threshold (ZOTUs; zero-radius OTUs). The dataset was further cleaned by removing sequences identified as chloroplasts or mitochondria, and by removing ZOTUs with less than 28 (0.001%) and 33 (0.001%) sequences for the 16S rRNA gene and ITS region datasets, respectively. The cleaned 16S rRNA gene and fungal ITS data were then rarefied at 5190 and 3019 sequences per sample, respectively.

### Statistical analysis

Statistical comparisons of ammonium and nitrate pools were performed in the R statistical environment [42] with a one-way ANOVA and a Tukey’s post-hoc with *p* value adjustment from the stats package [42]. Homogeneity of variance and normality were tested using the Levene’s and Shapiro–Wilk’s tests in the car [43] and stats packages, respectively. The data were transformed (cube root or log(x + 1)) in instances where they did not meet the required assumptions. For comparisons of microbial composition, the processed sequencing data were imported into the R statistical environment [42] and used to create a Phyloseq object [44]. To compare microbial composition between different recolonized soils, a Principal Coordinates Analysis (PCoA) with a Bray–Curtis dissimilarity index was used. Ordinations were performed using the ordinate function in the Phyloseq package. Patterns elucidated by ordination were tested statistically using Adonis (PERMANOVA) from the vegan package with 999 permutations [45]. To explore the influence of microbiome rediversification concentration (i.e. species richness) on microbial composition, we extracted Bray–Curtis dissimilarity values between the water control (i.e. Rec-WC) and the other microbiome rediversification treatments and plotted a linear regression using the ggpmisc package [46]. To identify nitrifying-associated taxa that were depleted and restored in our microcosms, we searched for genera within the families *Nitrospiraceae*, *Nitrosomonadaceae* and *Nitrococcaceae*. Of these families, the *Nitrospira*, *Nitrosospira*, *Ellin6067* (*Nitrosomonadaceae*) and *MND1* (*Nitrosomonadaceae*) genera were detected with a greater summed relative abundance of 0.1% across all microcosms. Taxa were grouped into restored (i.e. Rec-Fz, Rec-0 and Rec-2) and disrupted (i.e. Rec-6, Rec-8 and Rec-WC) ecological units to compare taxa. Grouping into ecological units was performed after considering our results, based on natural observed groupings in non-canonical ordination clustering and nitrate pool quantities (see Results), with the Rec-4 treatment included in the restored ecological unit for the farm soil and the disrupted ecological unit for the forest soil accordingly, and were statistically compared using a Kruskal–Wallis test in the stats package [42].

### Metagenomic analyses

**Fig. 2:**
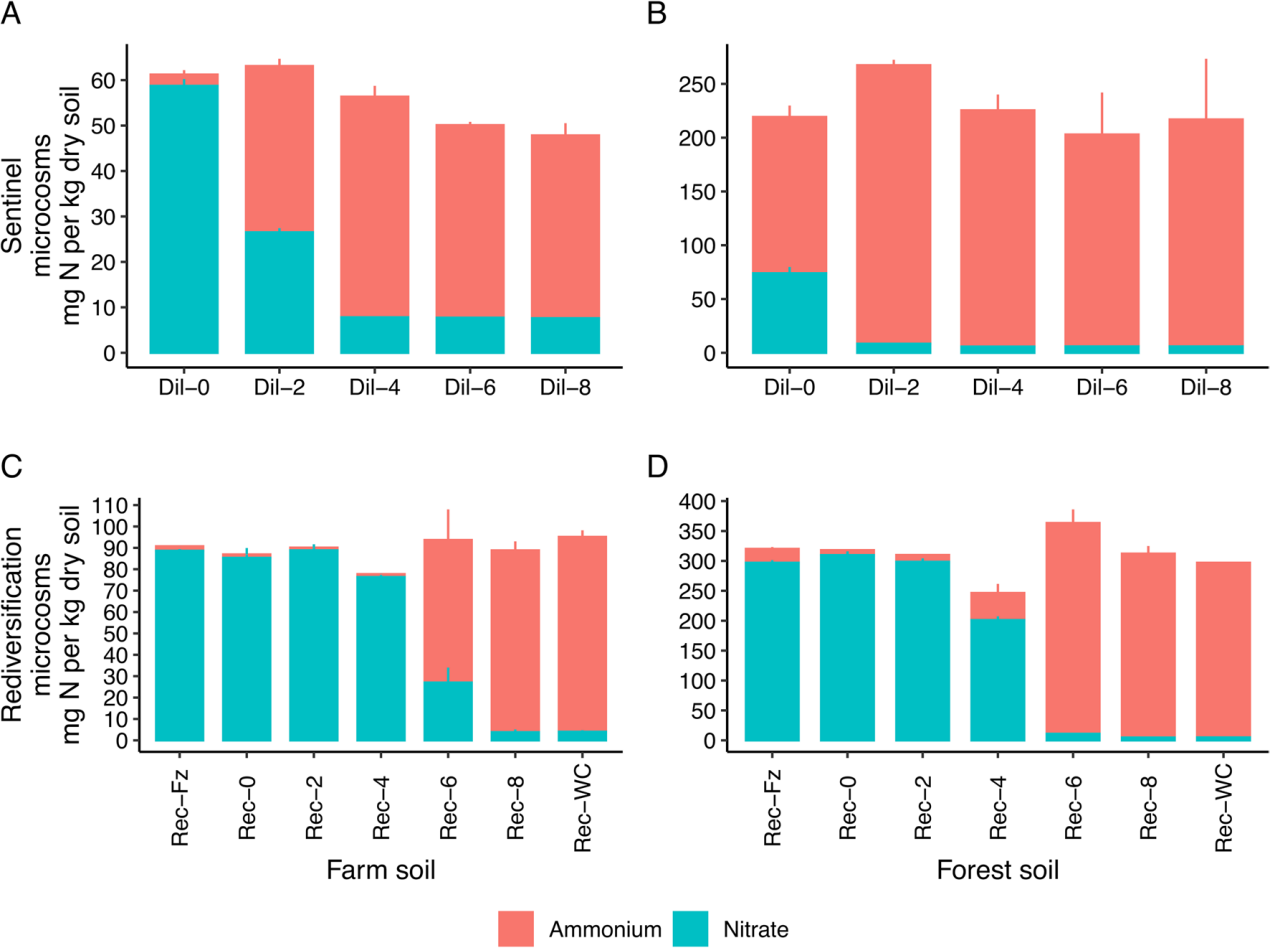
Loss and recovery of nitrification. Stacked bars of ammonium (red bars) and nitrate (blue bars) for sentinel (**A**, **B**) and rediversification (**C**, **D**) microcosms. Data are mean ± standard error. Note the differences in y-axis between the different soils and different microcosm experiments. See Additional file 1: Fig. S1 for non-stacked data

**Fig. 3:**
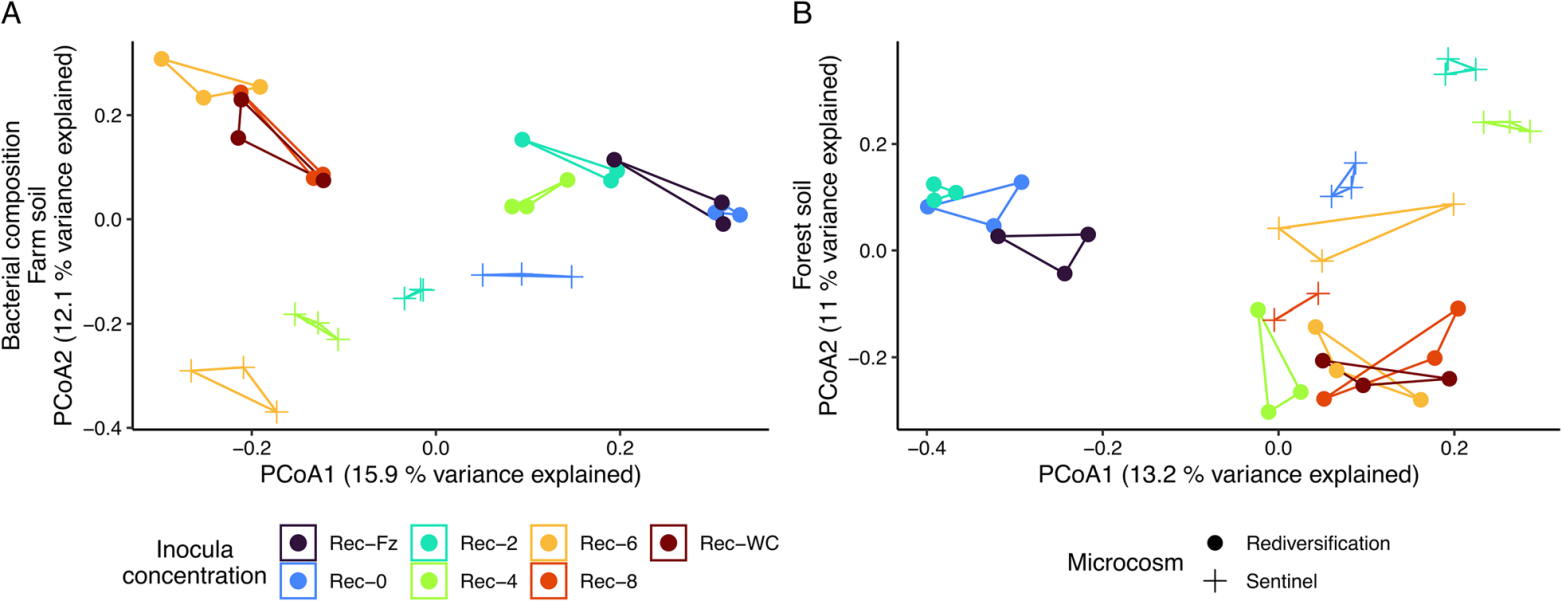
Principal Coordinate Analysis (PCoA) ordinations of bacterial compositions (16S rRNA gene). Panels **A** and **B** are the farm and forest soils, respectively. Bacterial compositions from the microbiome rediversification experiment (circle shapes) cluster according to nitrification status (see Fig. 2; i.e. with large and minor nitrate pools) into high (restored) and low (disrupted) concentration rediversification microcosms. PCoA ordinations of fungal compositions are displayed as Additional file 1: Fig. S2.

After determining that nitrification was disrupted and restored by manipulating soil biodiversity (see Fig. 2 in Results) and that microbial composition separated into disrupted and restored units (see Fig. 3 in Results), we chose to perform shotgun metagenomic sequencing from the Rec-0 and Rec-6 microcosms (total of 6 samples). We chose to sequence the Forest soil microcosms because of the greater effect of biodiversity manipulation on nitrification (see Fig. 2 in Results) We sought to identify differences in nitrogen metabolism between the two microcosms. Paired-ended shotgun metagenomic data were generated by the Pennsylvania State University Genomics Core Facility (Huck Institutes for the Life Sciences) on the NextSeq 2000 P2 device (150 × 150 bp). On average, we generated 4.6 gigabases of data for the Rec-0 microcosms and 3.7 gigabases of data for the Rec-6 microcosms. These values were greater than the sequencing depth recommended by Illumina (0.3 gigabases; “Shotgun Metagenomics Methods Guide” published in 2021) and a minimum recommended depth for best shotgun metagenomics practices (1 gigabase) [47]. Sequence quality was assessed using FastQC [48] and MultiQC [49]. Quality control, base correction, trimming and the removal of polyG tails from the metagenomic data were performed using fastp [50]. Trimmed metagenomic data were decontaminated against the human genome and human contaminants and had tandem repeats removed using Kneaddata, which uses Bowtie2 for decontamination [51]. Decontaminated data were imported into kbase [52] for further analysis. Taxonomic classification of cleaned reads was performed using GOTTCHA2 [53]. For the functional analysis, reads were assembled using MEGAHIT [54] and the functional annotation was performed using DRAM [55]. Assembly qualities were checked using QUAST [56].

## Results

### Reliable disruption and restoration of nitrification by manipulating microbial diversity

By manipulating microbial diversity through serial dilution, we observed a significant decrease in nitrate pools with decreasing microbial concentration in both soils for the sentinel microcosms (Farm soil: F_4,10_ = 1092, *p* < 0.001; Forest soil: F_4,10_ = 341, *p* < 0.001; Fig. 2; Additional file 1: Table S2). Importantly, nitrification was significantly disrupted in the sentinel “Dil-6” microcosms (i.e. 10^−6^) which acted as our foundation microcosms for the rediversification experiment. In the rediversification experiment, we observed significant differences in nitrate pools across diversity treatments for both soils (Farm soil: F_6,14_ = 107, *p* < 0.001; Forest soil F_6,14_ = 2302, *p* < 0.001; Additional file 1: Table S3). Microcosms that were inoculated with a high rediversification microbial concentration (i.e. the Rec-Fz, Rec-0 and Rec-2 microcosms) had significantly elevated nitrate pools relative to the treatments with minimal or no microbial addition (i.e. the Rec-6, Rec-8 and Rec-WC microcosms; Additional file 1: Table S3).

### Bacterial composition separated into disrupted and restored ecological units

Ordinations of bacterial composition identified discrete clustering of rediversification microcosms with high (i.e. restored; Rec-Fz, Rec-0 and Rec-2) and low (i.e. disrupted; Rec-6, Rec-8 and Rec-WC) microbial diversity additions (henceforth called restored and disrupted ecological units, respectively; Fig. 3), which corresponded with functional restoration (Fig. 2). We observed significant differences in bacterial composition according to the inoculum concentration in the farm (F_6,14_ = 3, R^2^ = 0.54, *p* ≤ 0.001) and forest soils (F_6,14_ = 3, R^2^ = 0.54, *p* ≤ 0.001). Discrete clustering was less clear for fungal compositions (Additional file 1: Fig. S2) and we only observed marginal significance between rediversification microcosms for the farm soil (F_6,14_ = 1, R^2^ = 0.37, *p* = 0.03).

### Functional restoration is associated with the re-introduction of nitrifying bacteria

**Fig. 4:**
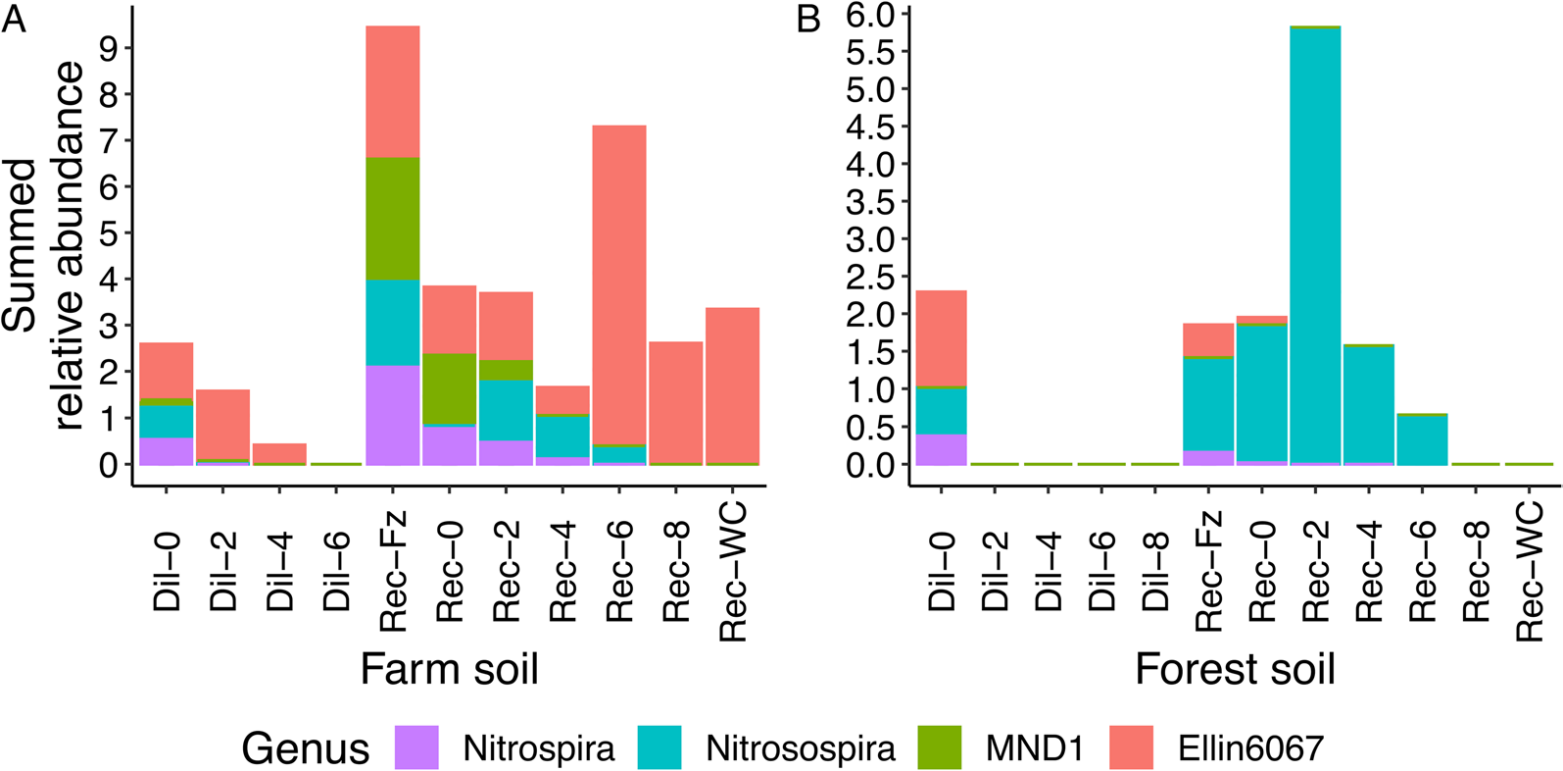
Summed relative abundance of taxa associated with nitrification. Panels **A** and **B** are the farm and forest soils, respectively. Additional taxa plots are shown as Additional file 1: Figs. S3 and S4.

In our dataset, we identified four genera associated with nitrification (Fig. 4). For our sentinel microcosms, we observed a greater relative abundance of the *Nitrospira* and *Nitrosospira* in both soils in our undiluted sentinel microcosms (Dil-0), and the *MND1* and *Ellin6067* genera were also overrepresented in the farm and forest soils, respectively (Additional file 1: Table S4). For the farm soil, we observed a greater relative abundance of the *Nitrospira*, *Nitrosospira* and *MND1* in our restored ecological unit microcosms and an overrepresentation of the *Ellin6067* in the disrupted ecological unit microcosms. For the forest soil, the *Nitrospira*, *Nitrosospira* and *Ellin6067* were overrepresented in our restored ecological unit microcosms.

### Regime shifts are stronger with greater bacterial richness

**Fig. 5:**
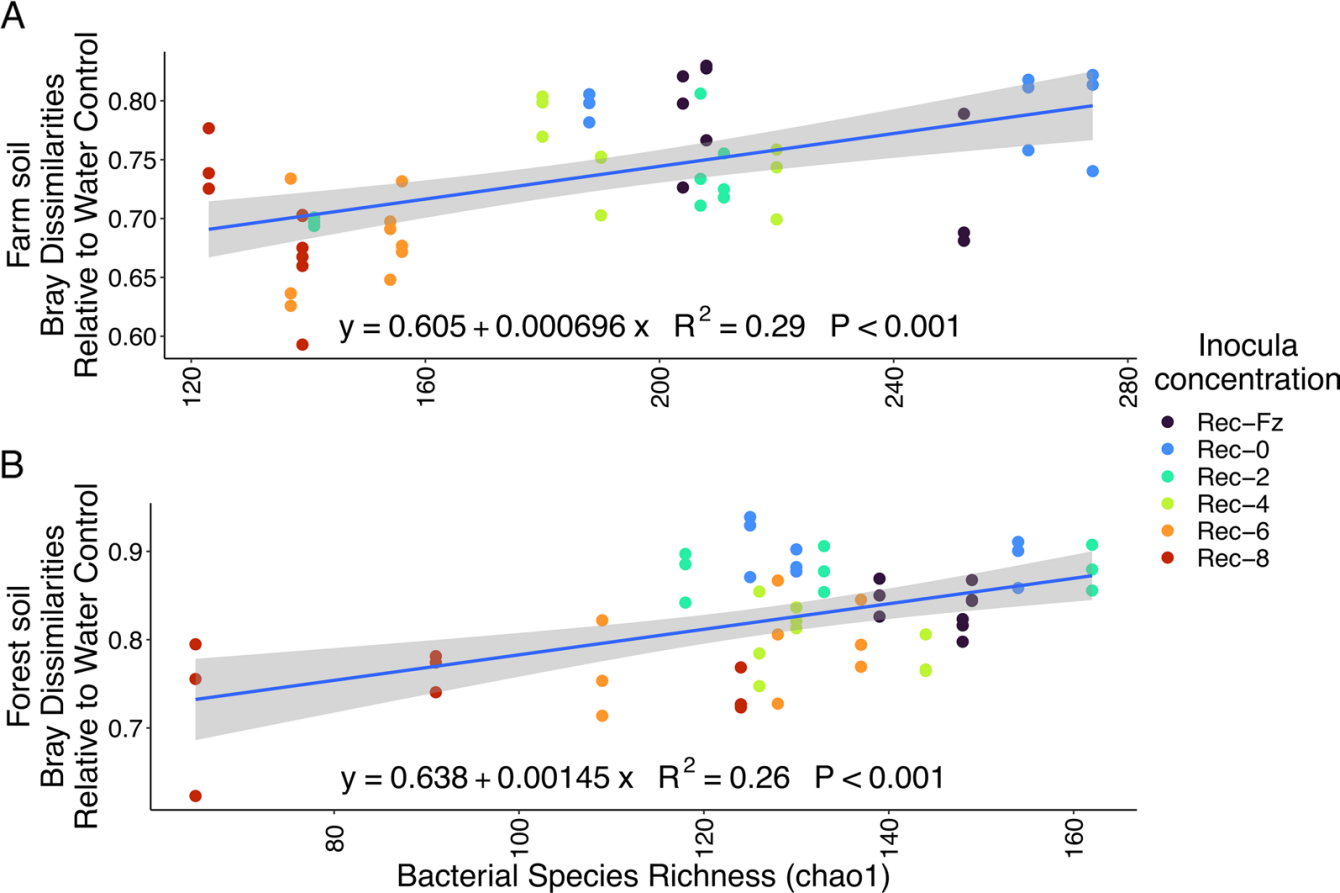
Linear trend between bacterial species richness and Bray dissimilarity to water control for bacterial composition. The gradient in bacterial species richness was generated by our microbiome rediversification concentration manipulations (color coded). Panels **A** and **B** are the farm and forest soils, respectively

To elucidate the influence of inoculum concentration on microbial community composition outcomes, we extracted Bray–Curtis dissimilarity values of our microbiome rediversification microcosms versus our water control microcosm for each soil (Additional file 1: Fig. S5). For both soils, fungal composition was more dissimilar to the water control than bacterial composition (Farm soil: H = 30, *d.f.* = 1, *p* < 0.001; Forest soil: H = 32, *d.f.* = 1, *p* < 0.001). We observed a significant linear relationship between species richness (Chao1) and Bray–Curtis dissimilarity to the water control (Fig. 5) for bacterial composition, with increasing dissimilarity to the water control corresponding to greater inocula concentrations. Only fungal composition in the farm soil showed a similar pattern to bacterial composition (Additional file 1: Fig. S6). In addition, examination of Bray–Curtis dissimilarity values within each rediversification treatment (i.e. between replicates; Additional file 1: Fig. S7) identified fungal composition as having greater within-treatment variability relative to bacterial composition (Farm soil: H = 42, *d.f.* = 1, *p* < 0.001; Forest soil: H = 31, *d.f.* = 1, *p* < 0.001).

### Disrupted microcosms are missing key nitrification enzymes and taxa

**Fig. 6:**
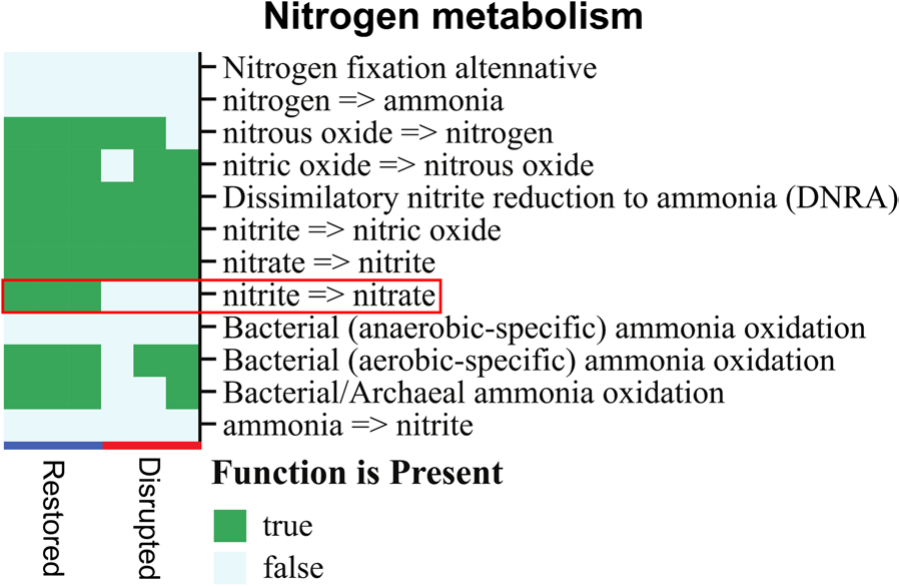
Nitrogen metabolism functional profile determined with shotgun metagenomic sequencing data and the DRAM functional profiler. Restored (Rec-0) and disrupted (Rec-6) microcosms from the Forest soil are shown. A complete functional profile is shown in Additional file 1: Figs. S8 and S9. The absence of nitrite-oxidation (red box) is observed in the disrupted microcosms

We performed shotgun metagenomic sequencing to characterize the functional potential of restored (i.e. Rec-0) and disrupted (i.e. Rec-6) microcosms, specifically to identify whether disruption of nitrification was also reflected in the nitrogen metabolism functional profile. The functional analysis identified the absence of enzyme-encoding genes that are ostensibly required for the conversion of nitrite to nitrate (*nxrA* and *nxrB*) in the disrupted microcosms (Fig. 6, Additional files 1: Figs. S8 and S9), which parallels the observations with the nitrate pool quantification (Fig. 2). Ammonia oxidation also appeared impacted, with *amoA* only identified in one disrupted microcosm and *hao* only detected in two disrupted microcosms. Species assigned as *Nitrobacter hamburgensis*, *N. vulgaris*, *Nitrosomonas communis*, *Nitrosospira briensis*, *N. lacus*, *N. multiformis* and *Nitrosovibrio tenuis* were detected in the restored microcosms, none of which were detected in the disrupted microcosms (Additional file 1: Table S5; Additional file 1: Fig. S10).

## Discussion

The ecological rescue of microbial functions using whole microbial assemblages is appealing, as soil functions are often provided by the additive contributions of many microbial taxa and existing microbial interactions can be helpful for enhancing overall community productivity [57]. Here, we sought to rescue soil function by reintroducing diverse microbial assemblages to microbially-depleted soils, focusing on nitrification as an example of functions that are both additive (i.e. performed by multiple microbial taxa) and sensitive to perturbation. We focus on the biotic constraints of microbiome rediversification. We show that microbial manipulations can impact nitrification, but that by reintroducing whole microbial assemblages, we can reliably rescue depleted function in two different soils.

In our microbiome rediversification experiment, our soil microcosms clearly separated into restored and disrupted ecological units according to nitrification restoration and bacterial composition. For both our soils in the microbiome rediversification experiment, we observed nitrification restoration when using the frozen soil (Rec-Fz), undiluted (Rec-0) and a two-fold serial dilution (Rec-2) microcosms as inocula, with the four-fold serial dilution (Rec-4) microcosm also restoring nitrification in the farm soil. As nitrification was restored in the Rec-2 microcosms, and the Rec-4 for the farm soil, it suggests that there was enough functional redundancy for nitrifying microorganisms to restore activity after substantial dilution of diversity. In support of our observations, denitrification activity was shown to be significantly impacted with serial dilutions of 10^−5^ in recolonized soils, [58], potential nitrification rates were significantly lower with dilutions of 10^−6^ [59] and nitrifiers may have densities between 10^4^ and 10^6^ cells per gram of soil [60]. While we did not directly quantify nitrifier diversity, bacterial species richness declines in the rediversification microcosms with reduced inoculum diversity (Additional file 1: Fig. S11), coupled with the significant decrease of nitrate and the lack of detectable nitrifying taxa and nitrite oxidation genes suggests the absence of nitrifying taxa or the strong interference of biotic constraints/interactions or the impact of community composition [61, 62] on nitrification. We also show the utility of using stored frozen soil as a microbial source, which matches our prior observations [33].

A previous study manipulated microbial diversity in an attempt to deplete and restore nitrification in soil, with diversity loss successfully disrupting nitrification [31]. Soils recolonized with diluted microbiomes (i.e. 10^−4^, 10^−6^ and 10^−8^) remained disrupted after 105 days, suggesting functional redundancy was not sufficient to maintain N-cycling equivalent to undiluted soil. Reintroductions of additional diluted microbiomes (i.e. 10^−4^ and 10^−6^) did not restore soil nitrification in their study. Two key differences between our study and their [31] study were: (1) the use of an intermediary soil and (2) the dilution concentrations (i.e. inocula diversity). Firstly, the intermediary soil used by Calderón et al. [31] was 0.7–1.3 pH units different from the pH in the initial soils used to inoculate the intermediary soil. As bacterial composition is strongly shaped by soil pH [63, 64], we would expect that the intermediary soil imparted significant abiotic pressures on the introduced soil bacteria, which may have inhibited functional restoration. In our design, we re-introduce microorganisms into their native soil to reduce the impact of abiotic constraints on microbial establishment. Secondly, Calderón et al. [31] used 10^−4^ as their highest diversity inoculum for microbiome rediversification, while we included higher microbial concentration treatments. In our data, we observed functional restoration in our Rec-2 (10^−2^) microcosms for both soils. A previous study had identified that N-cycling, specifically denitrification, is impaired with serial dilutions of 10^−5^ [58], it may be possible that the lack of restoration could be due to low microbial diversity, even after microbial additions.

A number of other studies have explored the impact of reducing microbial biodiversity on specialized soil functions (i.e., the diversity-function relationship) [34, 59, 61, 62, 65–67]. Two studies by Griffiths et al. [59, 65] observed the effect of biodiversity loss on potential nitrification rate [59] and nitrification [65] but inconsistent effects on a number of other soil functions. Wagg et al. [67] observed significant impacts on a number of ecosystem functions, including nitrogen transformation, after manipulating the diversity of different soil biota kingdoms with soil sieves of varying mesh sizes. Downing [61], Peters et al. [62] and Wagg et al. [67] highlighted the importance of community composition on ecosystem functions rather than species richness, while Trivedi et al. [34] and Singh et al. [66] showed specialized ecosystem functions were susceptible to diversity loss. Taken together, these studies suggest that specialized soil functions, such as various nitrogen transformations, can be disrupted by perturbation and changes in community composition. In our study, and in agreement with the previous studies, our microcosms separated into two distinct community compositions, the restored and disrupted ecological units, and we have indirect evidence of diversity loss. As an advance on the studies examining the diversity-function relationship, we made the additional step of reintroducing whole microbial assemblages to restore nitrification, and we used a recolonized water control (i.e., Rec-WC) as a contrasting treatment to show that microbiome rediversification is important for reintroducing key taxa.

We contrasted bacterial and fungal assembly patterns to determine how restoration attempts would differentially affect each microbial group. For bacterial composition, we observed a significant relationship between inocula species richness and the degree of community composition dissimilarity (i.e. a regime shift) in both soils, suggesting that the benefits of prior colonization may be dampened by the mass influx of bacteria. Additionally, bacterial composition had greater within-treatment similarity and greater similarity to the water control relative to fungal composition, suggesting a greater relative role for deterministic assembly. Meanwhile, fungal composition in microbially-inoculated treatments were more dissimilar to the water control and had lower within-treatment similarity relative to bacterial composition, suggesting that stochasticity may play a greater relative role in fungal assembly [68]. It should be noted that both deterministic and stochastic processes influence microbial assembly and it can be dependent on spatial scale, temporal influences and environmental factors [68–74]. When comparing the two soils, we observed similar assembly patterns for bacterial composition, but opposing patterns for fungal composition. We observed a significant, but weaker, relationship for fungal composition in the farm soil but no difference in the forest soil. These observations could be due to inadvertent fungal selection imparted in agricultural settings [68]. Recently, we observed that active fungal colonizer composition was strongly shaped across environments relative to bacterial colonizers [28] and agricultural settings often select for copiotrophic microorganisms [75]. It could be possible that a greater proportion of the farm soil fungal composition is copiotrophic in nature, explaining differences between the farm and forest soils.

Our study used a dilution-to-extinction approach to recolonize thrice autoclaved soils, similar to a previous study by Wagg et al. [67] albeit with only a single autoclave step. Autoclaving can break down soil nutrients and may impact other soil physicochemical properties [76] but allows for large-scale processing of soil for performing experiments at scale [28, 33, 74, 77, 78] and more closely resembles agricultural management practices such as soil steaming. We have previously observed greater unintended microbial regrowth in gamma irradiated soils relative to autoclaved soils (unpublished data). All forms of sterilization impact soil properties to an extent, but sterilized soils are a much more realistic simulated environment than solid or liquid culture. Different soil-types may respond differently to microbiome rediversification. In our data, we were able to rescue two different soil-types by using their own native microbiome. A previous study identified that the origin of the soil inoculum can steer restoration towards the donor ecosystem type [30]. We chose to reintroduce soil microbiomes into the sterile form of their native soil to restrict the abiotic constraints on microbial colonization. By reintroducing the native microbiome into their native soils, the microorganisms may have had a home-field advantage which could have facilitated an easier restoration of nitrification. Regardless, we did not observe a rescue of nitrification in our low diversity inoculum irrespective of the benefits of recolonizing a native soil.

## Conclusions

Many soil functions are additive in nature and require numerous taxa to functionally contribute to individual pathway steps. Manipulating microbiome composition is an appealing concept, as it can retain existing microbial relationships and lessen the impact of biotic constraints during microbial delivery in agrosystems. Here, we manipulated microbial assemblages to disrupt and rescue N-cycling in two soils. Our data indicated that microbial diversity could be manipulated to disrupt nitrification and that microbial consortia could be reintroduced to restore nitrification. Bacterial composition separated into disrupted and restored ecological units, which also corresponded with the presence of key N-cycling microorganisms and their associated enzymes, indicating the impact of species loss, community composition and/or biotic constraints on the rescue of nitrification. We also investigated how existing residents could impact the establishment of microbial consortia with differing degrees of diversity, and we identified a significant positive relationship between inoculum diversity and Bray–Curtis dissimilarity to a water microbiome control for bacteria. We have shown that additive soil functions can be disrupted and rescued by manipulating soil microbial diversity. These data highlight the plausibility of manipulating microbial assemblages to disrupt and rescue functions of interest in soil and the importance of biotic constraints for microbial establishment and functional contribution.

## Supplementary Information

**Additional file 1.** Table S1.

## Data Availability

Raw data files in FASTQ format were deposited in the NCBI sequence read archive under BioProject number PRJNA850792.
