# Supplementary material for: Leveraging microbiome rediversification for the ecological rescue of soil function
Source: Environ Microbiome. 2023 Jan 23;18:7. doi: 10.1186/s40793-023-00462-4 (PMC9872425; doi:10.1186/s40793-023-00462-4)
Supplement: Supplementary file 1 — Additional file 1. Table S1. [file 40793_2023_462_MOESM1_ESM.docx]

Leveraging microbiome rediversification for the ecological rescue of soil function

William L King^1,2^, Sarah C Richards^1,3,4^, Laura M Kaminsky^1^, Brosi A Bradley^5^, Jason P Kaye^3,5^, Terrence H Bell^1,3,4*^

^1^Department of Plant Pathology and Environmental Microbiology, The Pennsylvania State University, University Park, PA, 16802, USA

^2^School of Integrative Plant Science, Cornell University, Ithaca, NY, 14853, USA

^3^Intercollege Graduate Degree Program in Ecology, The Pennsylvania State University, University Park, PA, 16802, USA

^4^Intercollege Graduate Degree Program in International Agriculture and Development, The Pennsylvania State University, University Park, PA, 16802, USA

^5^Department of Ecosystem Science and Management, The Pennsylvania State University, University Park, PA, 16802, USA

*Correspondence:

Postal address: The Pennsylvania State University, 317 Buckhout Lab, University Park, PA, USA, 16802

Telephone number: 814-865-9653

Email: terrence.bell@utoronto.ca

Table S1: Soil analysis. Provided data includes pH, total nitrogen (N), total carbon (C), phosphorous (P), potassium (K), magnesium (Mg), calcium (Ca), organic matter (OM), zinc (Zn), copper (Cu) and sulfur (S). All units are parts per million (ppm) unless specified.

| Soil | pH | N (%) | C (%) | P | K | Mg | Ca | OM (%) | Zn | Cu | S | Textural class |
| --- | --- | --- | --- | --- | --- | --- | --- | --- | --- | --- | --- | --- |
| Sterile Farm | 6.7 | 0.12 | 1.35 | 31 | 135 | 101 | 1192 | 1.8 | 1.8 | 2.2 | 11.7 | Silt Loam |
| Sterile Forest | 5.7 | 0.51 | 5.93 | 31 | 114 | 171 | 831 | 10.0 | 3.7 | 0.6 | 20.3 | Sandy Loam |

Figure S1: Nitrogen pools for the sentinel and microbiome rediversification microcosms. Note the y-axis differences. Capital letters are for ammonium comparisons and lowercase letters are nitrate comparisons. Shown comparisons are for comparisons against the undilute (Dil-0 and Rec-0) and Dil-4/Rec-4 microcosms corresponding to Supplementary Tables 2 and 3.

Table S2: Comparisons of ammonium and nitrate pools for the sentinel microcosms.

| Soil | Overall | Dilution | Comparison | Ammonium | Nitrate |
| --- | --- | --- | --- | --- | --- |
| Farm | Ammonium:  F_4,10_ = 116, p < 0.001  Nitrate:  F_4,10_ = 1092, p < 0.001 | Dil-0 | Dil-2  Dil-4  Dil-6  Dil-8 | q < 0.001  q < 0.001  q < 0.001  q < 0.001 | q < 0.001  q < 0.001  q < 0.001  q < 0.001 |
|  |  | Dil-2 | Dil-4  Dil-6  Dil-8 | NS  NS  NS | q < 0.001  q < 0.001  q < 0.001 |
|  |  | Dil-4 | Dil-6  Dil-8 | NS  NS | NS  NS |
|  |  | Dil-6 | Dil-8 | NS | NS |
| Forest | Ammonium:  NS  Nitrate:  F_4,10_ = 341, p < 0.001 | Dil-0 | Dil-2  Dil-4  Dil-6  Dil-8 | NA  NA  NA  NA | q < 0.001  q < 0.001  q < 0.001  q < 0.001 |
|  |  | Dil-2 | Dil-4  Dil-6  Dil-8 | NA  NA  NA | NS  NS  NS |
|  |  | Dil-4 | Dil-6  Dil-8 | NA  NA | NS  NS |
|  |  | Dil-6 | Dil-8 | NA | NS |

Table S3: Comparisons of ammonium and nitrate pools for the microbiome rediversification microcosms.

| Soil | Overall | Dilution | Comparison | Ammonium | Nitrate |
| --- | --- | --- | --- | --- | --- |
| Farm | Ammonium:  F_6,14_ = 287,  p < 0.001  Nitrate:  F_6,14_ = 107,  p < 0.001 | Rec-0 | Rec-Fz  Rec-2  Rec-4  Rec-6  Rec-8  Rec-WC | NS  NS  NS  q < 0.001  q < 0.001  q < 0.001 | NS  NS  NS  q < 0.001  q < 0.001  q < 0.001 |
|  |  | Rec-4 | Rec-Fz  Rec-0  Rec-2  Rec-6  Rec-8  Rec-WC | NS  NS  NS  q < 0.001  q < 0.001  q < 0.001 | NS  NS  NS  q < 0.001  q < 0.001  q < 0.001 |
| Forest | Ammonium:  F_6,14_ = 190,  p < 0.001  Nitrate:  F_6,14_ = 2302,  p < 0.001 | Rec-0 | Rec-Fz  Rec-2  Rec-4  Rec-6  Rec-8  Rec-WC | NS  NS  NS  q < 0.001  q < 0.001  q < 0.001 | NS  NS  q < 0.001  q < 0.001  q < 0.001  q < 0.001 |
|  |  | Rec-4 | Rec-Fz  Rec-0  Rec-2  Rec-6  Rec-8  Rec-WC | NS  NS  NS  q < 0.001  q < 0.001  q < 0.001 | q < 0.001  q < 0.001  q < 0.001  q < 0.001  q < 0.001  q < 0.001 |

Figure S2: Principal Coordinate Analysis (PCoA) ordinations of fungal compositions (ITS region). Plus shapes refer to the sentinel microcosms and solid circles refer to the microbiome rediversification microcosms.

Table S4: Comparisons of individual nitrogen cycling genera. Taxa were grouped into restored (i.e. Rec-Fz, Rec-0 and Rec-2) and disrupted (i.e. Rec-6, Rec-8 and Rec-WC) ecological units for comparison with a Kruskal test.

| Soil | Microcosm | *Nitrospira* | *Nitrosospira* | *MND1* | *Ellin6067* |
| --- | --- | --- | --- | --- | --- |
| Farm | Sentinels | H = 11,  *d.f*. = 1  p = 0.001 | H = 4,  *d.f*. = 1  p = 0.04 | H = 7,  *d.f*. = 1  p = 0.01 | NS |
|  | Rediversification | H = 10,  *d.f*. = 1  p = 0.001 | H = 5,  *d.f*. = 1  p = 0.02 | H = 9,  *d.f*. = 1  p = 0.003 | H = 6,  *d.f*. = 1  p = 0.02 |
| Forest | Sentinels | H = 13,  *d.f*. = 1  p < 0.001 | H = 13,  *d.f*. = 1  p < 0.001 | NS | H = 8,  *d.f*. = 1  p = 0.005 |
|  | Rediversification | H = 4,  *d.f*. = 1  p = 0.04 | H = 9,  *d.f*. = 1  p = 0.003 | NS | H = 8,  *d.f*. = 1  p = 0.004 |

Figure S3: Bacterial taxa plots summarized at the Class level. Data are averaged relative abundance. The inocula concentration is shown for both sentinel microcosms (i.e. Dil-0 to Dil-8) and microbiome diversification microcosms (i.e. Rec-Fz to Rec-WC).

Figure S4: Fungal taxa plots summarized at the Class level. Data are averaged relative abundance. The inocula concentration is shown for both sentinel microcosms (i.e. Dil-0 to Dil-8) and microbiome diversification microcosms (i.e. Rec-Fz to Rec-WC).

Figure S5: Bray-Curtis dissimilarity values of the microbiome rediversification microcosms relative to the water control microcosm for each soil. Bacterial and fungal compositions are circles and triangles, respectively. Data are mean ± standard error.

Figure S6: Linear trend between fungal species richness and dissimilarity to water control for bacterial composition. The gradient in fungal species richness was generated by our microbiome rediversification concentration manipulations (color coded).

Figure S7: Within microbiome rediversification treatment variance. Data are Bray-Curtis dissimilarity values between replicates within each treatment for both bacterial (circles) and fungal (triangles) compositions.

Figure S8: Electron transport chain functional profile determined with shotgun metagenomic sequencing data and the DRAM functional profiler. Restored (Rec-0) and disrupted (Rec-6) microcosms from the Forest soil are shown.

Figure S9: Functional profile determined with shotgun metagenomic sequencing data and the DRAM functional profiler. Restored (Rec-0) and disrupted (Rec-6) microcosms from the Forest soil are shown.

Table S5: Relative abundance of bacterial species associated with nitrogen metabolism from shotgun metagenomics data.

| Species | Restored replicate 1 | Restored replicate 2 | Restored replicate 3 | Disrupted replicate 1 | Disrupted replicate 2 | Disrupted replicate 3 |
| --- | --- | --- | --- | --- | --- | --- |
| *Nitrobacter hamburgensis* | 0.02 % | 0.005 % | 0.03 % | 0 % | 0 % | 0 % |
| *Nitrobacter vulgaris* | 0.006 % | 0.002 % | 0.007 % | 0 % | 0 % | 0 % |
| *Nitrosomonas communis* | 0.001 % | 0 % | 0 % | 0 % | 0 % | 0 % |
| *Nitrosospira briensis* | 0.004 % | 0.0006 % | 0.005 % | 0 % | 0 % | 0 % |
| *Nitrosospira lacus* | 0.008 % | 0.001 % | 0.01 % | 0 % | 0 % | 0 % |
| *Nitrosospira multiformis* | 0.008 % | 0.002 % | 0.01 % | 0 % | 0 % | 0 % |
| *Nitrosovibrio tenuis* | 0.003 % | 0 % | 0.004 % | 0 % | 0 % | 0 % |

Figure S10: Class-level bacterial compositions of Restored (Rec-0) and Disrupted (Rec-6) microcosms. Data were generated from shotgun metagenomic sequencing data and individual replicates are shown.

Figure S11: Bacterial species richness for the sentinel and rediversification microcosms. Data are separated into the Farm and Forest soil types.
